# Combination of Total Psoas Index and Albumin–Globulin Score for the Prognosis Prediction of Bladder Cancer Patients After Radical Cystectomy: A Population-Based Study

**DOI:** 10.3389/fonc.2021.724536

**Published:** 2021-09-20

**Authors:** Keyi Wang, Yongzhe Gu, Jinliang Ni, Houliang Zhang, Jinbo Xie, Tianyuan Xu, Jiang Geng, Weipu Mao, Bo Peng

**Affiliations:** ^1^Department of Urology, Shanghai Shidong Hospital of Yangpu District, Shanghai, China; ^2^Department of Urology, Shanghai Tenth People’s Hospital, School of Medicine, Tongji University, Shanghai, China; ^3^Department of Neurology, Shanghai Tenth People’s Hospital, School of Medicine, Tongji University, Shanghai, China; ^4^Department of Urology, Tenth People’s Hospital, Anhui Medical University, Shanghai, China; ^5^Department of Urology, Affiliated Zhongda Hospital of Southeast University, Nanjing, China

**Keywords:** bladder cancer, prognostic predictor, albumin–globulin score, total psoas index, nanogram

## Abstract

**Background:**

Sarcopenia as the loss of skeletal muscle mass is related with poor postoperative survival. This work purposed to evaluate the prognostic prediction of the total psoas index (TPI), albumin–globulin score (AGS), and the combination of TPI and AGS (CTA) in bladder cancer (BCa) patients after radical cystectomy.

**Methods:**

BCa patients that received radical cystectomy between 2012 and 2020 were retrieved from our medical center. The calculation of TPI was based on the plain computed tomography images. The predictive effects of TPI, AGS, and CTA grade on survival of BCa patients were analyzed and compared with the albumin–globulin ratio (AGR) through the receiver operating characteristic (ROC) curves. A nomogram was further established based on the Cox regression results from CTA grade and clinicopathological characteristics, which are verified by the decision curve analysis (DCA).

**Results:**

A total of 112 eligible patients diagnosed as BCa were included in this study for retrospective analysis. The patients with lower TPI or higher AGS grade (1/2) contained poorer overall survival (OS) and disease-free survival (DFS). Divided by CTA grade, there were 35 (31.25%) patients in grade 1 associated with the best postoperative prognosis, which was accompanied with increased TPI and decreased AGS. The CTA grade could better predict postoperative outcomes compared with TPI, AGR, and AGS for the highest area under the curve (AUC; 0.674 of OS and 0.681 of DFS). The 3- and 5-year OS and DFS nomograms were conducted based on CTA grade and clinical variables, with a higher predictive performance than the TNM stage.

**Conclusion:**

This study revealed that the novel index CTA functioned as an effective prognostic predictor for postoperative OS and DFS of BCa patients after radical cystectomy. Preoperative assessment of CTA would contribute to optimizing clinical therapies.

## Introduction

Bladder cancer (BCa) is considered as one of the most malignant tumors without promising progress in the treatment for 30 years (1, 2). It was estimated that 81,400 cases of BCa will be diagnosed in the USA in 2020, with 33,820 deaths (3). Surgery is the most effective treatment for BCa patients, and radical cystectomy (RC), the standard for invasive bladder cancer, has been worldly applied (4). For the accurate prognosis prediction, a series of indicators were introduced to predict the overall survival (OS) and disease-free survival (DFS) of BCa patients after RC, including TNM stage and marital status (5–7). A simple and effective predictive factor or model for the prognosis of patients assists clinicians to establish the comprehensive individualized treatment of BCa, which will expand the survival time and quality.

In the recent decades, inflammation-related models such as lymphocyte/monocyte ratio (LMR), neutrophil to lymphocyte ratio (NLR), and systemic immune-inflammation index (SII) have been used to evaluate the survival of patients with various tumors (8, 9). The inflammation responses and systemic dystrophia enhanced tumor angiogenesis and invasion through affecting the tumor microenvironment and attenuating immunologic function (10). Albumin (ALB) and globulin (GLB), the crucial parts of human serum proteins, have been considered as independent prognostic predictors for patients among kinds of cancers (11, 12) due to the regulation of inflammatory cytokines. The albumin-to-globulin ratio (AGR) and albumin–globulin score (AGS), the indicators based on ALB and GLB, have been proved to function well in the prognosis evaluation of lung cancer and liver tumor (10, 13). However, there were no reports assessing the association between AGS with postoperative long-term outcomes of BCa patients after RC.

Sarcopenia, a geriatric syndrome, is characterized by the loss of physical function and skeletal muscle mass with increasing age (14, 15). The occurrence of sarcopenia was observed in patients with inflammatory diseases, frailty, and malignancies (16). There were studies indicating that the total psoas index (TPI) for sarcopenia evaluation predicts well the survival of BCa patients after RC (15, 17). The comprehensive assessment of the systemic inflammatory response and nutritional status will play an important role in the long-term survival prediction of BCa patients after RC. This study aimed to evaluate the predictive effect of AGS and TPI on the prognosis of postoperative BCa patients, simultaneously compared with AGR. Next, a unique index from the combination of total psoas index and albumin–globulin score (CTA) was represented to comprehensively assess the inflammatory and nutritional condition of patients. Furthermore, a nomogram based on CTA was established for exploration of CTA-grade impacts on long-term outcomes of BCa patients.

## Material and Methods

### Patients

In this retrospective study, a total of 112 BCa patients who underwent RC were enrolled at Shanghai Tenth People’s Hospital between January 2012 and December 2020. The exclusion criteria were as follows: patients with a history of malignancies; psychiatric abnormalities; without complete experimental data; and missing follow-up data. Written informed consent was provided by all patients. This study was approved by the Medical Ethics Committee of Shanghai Tenth People’s Hospital (SHSY-IECKY-4.0/18-68/01) in adherence to the guidelines of the 1975 Helsinki Declaration.

### Clinical Variables and Follow-Up

All the information of patients was collected from the hospital electronic records or handwritten records. The following clinical factors were collected: age, gender, body mass index (BMI), Charlson Comorbidity Index (CCI), adjuvant chemotherapy, tumor-node metastasis (TNM) stages, tumor grade, and hemoglobin. The optimal cutoff values of ALB, GLB AGR, and TPI were 34.02 g/l, 26.13 U/l, 1.52, and 540.60 mm^2^/m^2^, respectively. The AGR was calculated as ALB level divided by serum GLB level. The definition of AGS was as follows: both normal values of ALB (>34.02 g/l) and GLB (≤26.13 g/l) were assigned to AGS of 0, decreased ALB (≤34.02 g/l) and elevated GLB (>26.13 g/l) were stratified to AGS of 2, and one of the abnormal values of ALB and GLB was divided to AGS of 3 (13). The calculation of TPI was based on the plain computed tomography images. Moreover, the value of TPI is calculated based on the cross-sectional area from the two total psoas area (TPA) muscles which pass through the third lumbar (L3) cone. The value is further standardized by the patients’ height: TPA/(height [m] × height [m]). Then, the CTA grade was classified as the following: both decreased AGS (0) and elevated TPI patients were stratified into CTA grade 1, both high AGS (1 or 2) and decreased TPI patients were classified into CTA grade 3, and the others patients were divided into CTA grade 2. Moreover, the hospital time and the survival time of patients were acquired. Each patient was followed up regularly after surgery, every 3 months for the first 2 years, and then every 6 months thereafter. Information of each patient was registered including tumor recurrence after surgery and survival status. Overall survival (OS) was calculated from received RC to death or to the last follow-up date. Disease-free survival (DFS) was calculated from date of RC to disease recurrence or to the date of the last follow-up.

### Statistical Analysis

The software of SPSS (version 22.0, Chicago, IL, USA), MedCalc (version 15.2.2.0, Ostend, Belgium), and GraphPad Prism (version 8.0, San Diego, CA, USA) were used to perform the statistical analyses. The optimal cutoff values of ALB, GLB, AGR, and TPI were determined by receiver-operating characteristic (ROC) curves with 2-year survival as the end point. According to each cutoff value, overall survival curves (OS) and disease-free survival curves (DFS) were compared by Kaplan–Meier survival analysis and the log-rank test was applied to test the differences. The area under the curve (AUC) of CTA, AGS, AGR, and TPI was measured and compared. Potentially related risk factors were identified through the univariate Cox proportional hazard regression. Furthermore, clinicopathological factors (*p <*0.2) of the results were proceeded to multivariable Cox regression to confirm the independent prognostic factors for OS and DFS. R software version 3.5.1 (http://www.R-project.org) was conducted for establishing nomograms based on the multivariable Cox regression results. The R packages applied in this work were “rmda” and “rms”. Calibration curves were measured to evaluate the predictive performance of nomograms with the calibration curve agreed, with a 45° line indicating the perfect predictive accuracy.

## Results

### Patient Baseline Characteristics

One hundred twelve eligible BCa patients receiving surgical resection were included in this study from 2012 to 2020 in Shanghai Tenth People’s Hospital. The baseline characteristics were summed up in **Table 1**. The median age was 65.63 years, and there were 97 (86.6%) male patients. For the eligible patients, their median BMI was 23.51 ± 3.06 kg/m^2^ and the TPI was 538.52 ± 73.44 mm^2^/m^2^. There were 64 (57.1) patients divided into the CCI ≤ 2 group. Serum factors including hemoglobin, ALB, and GLB were 121.38 ± 20.74 g/l, 38.62 ± 6.61 g/l, and 25.36 ± 4.22 U/l, separately. Moreover, the AGR of the included patients was 1.56 ± 0.35. The postoperative mean OS was survival time of 41.44 ± 27.01 months for the BCa patient after RC.

**Table 1:** Baseline characteristics of included BCa patients.

Variables	No. (%)
Total patients	112 (100.0)
Age [year, mean (SD)]	65.63 (10.66)
Age categorized	
≤65	56 (50.0)
>65	56 (50.0)
Gender	
Male	97 (86.6)
Female	15 (13.4)
BMI [kg/m^2^, mean (SD)]	23.51 (3.06)
BMI categorized [kg/m^2^]	
<18.5	4 (3.5)
18.5~23.9	60 (53.5)
24.0~26.9	37 (33.0)
≥27.0	10 (9.0)
CCI	
≤2	64 (57.1)
>2	48 (42.8)
Adjuvant chemotherapy	
No	91 (81.2)
Yes	21 (18.8)
T-stage	
T1	46 (41.0)
T2	21 (18.7)
T3	24 (21.4)
T4	21 (18.7)
N-stage	
N0	95 (84.8)
N1	17 (15.2)
M-stage	
M0	108 (96.4)
M1	4 (3.6)
AJCC grade	
Low grade	8 (7.1)
High grade	104 (92.9)
Hemoglobin [g/L, mean (SD)]	121.38 (20.74)
ALB [g/L, mean (SD)]	38.62 (6.61)
GLB [U/L, mean (SD)]	25.36 (4.22)
AGR [mean, (SD)]	1.56 (0.35)
TPI [mm^2^/m^2^, (SD)]	538.52 (73.44)
Hospital time (days)	33.52 (9.14)
Survival time (months)	41.44 (27.01)

BCa, bladder cancer; BMI, body mass index; CCI, Charlson Comorbidity Index; SD, standard deviation; AJCC, American Joint Committee on Cancer; ALB, albumin; GLB, globulin; AGR, albumin to globulin ratio; TPI, total psoas index; TNM, tumor-node metastasis.

### Clinical Characteristics According to TPI, AGS, and CTA

**Figure 1:**
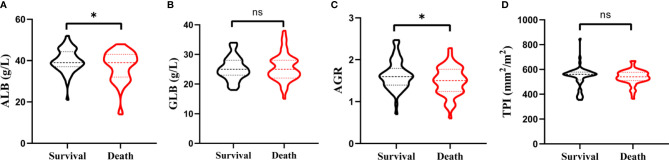
Violin plots exhibiting the preoperative ALB **(A)**, GLB **(B)**, AGR **(C)**, and TPI **(D)** level in subgroups of survival and death. ns represents no statistical difference and *p < 0.05.

As shown in **Figure 1**, the distributions of the ALB, GLB, AGR, and TPI levels for patients before RC were divided by survival outcomes. After stratification, the patients’ characteristics were demonstrated based on TPI and AGS (**Table 2**). There were 67 (59.82%) patients containing high TPI with a mean BMI of 24.71 kg/m^2^. Among them, 64 (95.5%) were in the M0 group and 65 (97.0%) were of high AJCC grade. The hemoglobin was higher than that in the low TPI group (124.76 ± 19.12 vs. 116.36 ± 22.25 g/l; *p* = 0.035). The mean ALB, GLB, and AGR were 38.85 g/l, 25.39 U/l, and 1.57, respectively. For postoperative OS time, the high TPI group was better than the low group (42.66 ± 5.04 vs. 39.64 ± 9.90 m; *p* = 0.038).

**Table 2:** Baseline characteristics according to the subgroups classified by TPI and AGS.

Characteristic	TPI		P value	AGS		P value
	Low (0)	High (1)		Low (0)	High (1/2)	
	No. (%)	No. (%)		No. (%)	No. (%)	
Total patients	45 (40.17)	67 (59.82)		55 (49.10)	57 (50.89)	
Age [year, mean (SD)]	67.82 (10.77)	64.16 (10.42)	0.075	64.13 (10.79)	67.09 (10.44)	0.143
Age categorized			0.177			0.571
≤65	19 (42.2)	37 (55.2)		29 (52.7)	27 (47.4)	
>65	26 (57.8)	30 (44.8)		26 (47.3)	30 (52.6)	
Gender			<0.001			0.725
Male	30 (66.7)	67 (100)		47 (85.5)	50 (87.7)	
Female	15 (33.3)	0 (0)		8 (14.5)	7 (12.3)	
BMI [kg/m^2^, mean (SD)]	21.73 (2.56)	24.71 (2.80)	<0.001	23.77 (2.94)	23.26 (3.20)	0.388
BMI categorized, kg/m^2^			0.001			0.496
<18.5	4 (8.9)	1 (1.5)		2 (3.6)	3 (5.3)	
18.5~23.9	30 (66.7)	30 (44.8)		26 (47.3)	34 (59.6)	
24.0~26.9	11 (24.4)	26 (38.8)		21 (38.2)	16 (28.1)	
≥27.0	0 (0)	10 (14.9)		6 (10.9)	4 (7.0)	
CCI			0.911			0.585
≤2	26 (57.8)	38 (56.7)		30 (54.5)	34 (59.6)	
>2	19 (42.2)	29 (43.3)		25 (45.5)	23 (40.4)	
Adjuvant chemotherapy			0.478			0.880
No	38 (84.4)	53 (79.1)		45 (81.8)	46 (80.7)	
Yes	7 (15.6)	14 (20.9)		10 (18.2)	11 (19.3)	
T-stage			0.335			0.097
T1	19 (42.2)	27 (40.3)		27 (49.1)	19 (33.3)	
T2	9 (20.0)	12 (17.9)		12 (21.8)	9 (15.8)	
T3	12 (26.7)	12 (17.9)		10 (18.2)	14 (24.6)	
T4	5 (11.1)	16 (23.9)		6 (10.9)	15 (26.3)	
N-stage			0.128			0.022
N0	41 (91.1)	54 (80.6)		51 (92.7)	44 (77.2)	
N1	4 (8.9)	13 (19.4)		4 (7.3)	13 (22.8)	
M-stage			0.648			0.359
M0	44 (97.8)	64 (95.5)		52 (94.5)	56 (98.2)	
M1	1 (2.2)	3 (4.5)		3 (5.5)	1 (1.8)	
AJCC grade			0.058			1.000
Low grade	6 (13.3)	2 (3.0)		4 (7.3)	4 (7.0)	
High grade	39 (86.7)	65 (97.0)		51 (92.7)	57 (93.0)	
Hemoglobin (g/L), mean (SD)	116.36 (22.25)	124.76 (19.12)	0.035	127.35 (18.50)	115.63 (21.32)	0.002
ALB [g/L, mean (SD)]	38.29 (7.87)	38.85 (5.66)	0.661	40.78 (3.74)	36.54 (8.01)	0.001
GLB [U/L, mean (SD)]	25.33 (4.73)	25.39 (0.38)	0.947	22.85 (2.19)	27.79 (4.30)	<0.001
AGR [mean, (SD)]	1.55 (0.38)	1.57 (0.33)	0.768	1.80 (0.26)	1.32 (0.25)	<0.001
Hospital time (days)	35.54 (8.36)	32.16 (9.45)	0.055	33.56 (9.09)	33.48 (9.27)	0.963
Survival time (months)	39.64 (9.90)	42.66 (5.04)	0.038	26.99 (3.64)	25.97 (3.44)	0.021

BMI, body mass index; CCI, Charlson Comorbidity Index; SD, standard deviation; AJCC, American Joint Committee on Cancer; ALB, albumin; GLB, globulin; AGR, albumin to globulin ratio; TPI, total psoas index; AGS, albumin–globulin score; TNM, tumor-node metastasis.

**Figure 2:**
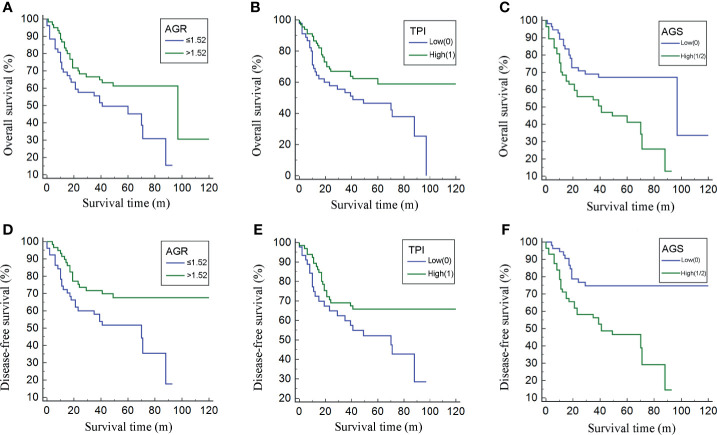
Kaplan–Meier curves for overall survival (OS) and disease-free survival (DFS) divided by albumin to globulin ratio (AGR) **(A, D)**, albumin–globulin score (AGS) **(B, E)**, and total psoas index (TPI) **(C, F)**.

Additionally, the patients were also divided by AGS and 55 (49.10%) were stratified into the low AGS group with mean BMI of 23.77 kg/m^2^. The serum ALB value was higher (40.78 ± 3.74 vs. 36.54 ± 8.01 g/l; *p* = 0.001), while the GLB value was decreased (22.85 ± 2.19 vs. 27.79 ± 4.30 U/l; *p* < 0.001) in the low AGS group. As for AGR, the value was higher in the low AGS group (1.80 ± 0.26 vs. 1.32 ± 0.25; *p* < 0.001). Moreover, the OS was significantly higher in the low AGS group (26.99 ± 3.64 m) than in the high AGS group (25.97 ± 3.44 m) (*p* = 0.021). Kaplan–Meier survival curves exhibited that patients with high AGR (>1.52) were related with greater OS (*p* = 0.036, **Figure 2A**) and DFS (*p* = 0.016, **Figure 2D**). Patients in the high TPI group contained better OS (*p* = 0.045, **Figure 2B**) and DFS (*p* = 0.049, **Figure 2E**). Moreover, patients in the low AGS group were associated with longer OS (*p* = 0.002, **Figure 2C**) and DFS (*p* < 0.001, **Figure 2F**).

**Figure 3:**
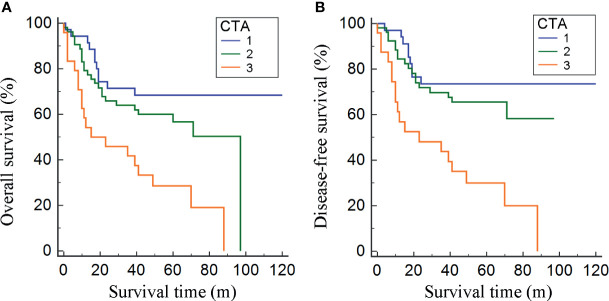
Kaplan–Meier curves for OS and DFS divided by the combination of total psoas index and albumin–globulin score (CTA) grade **(A, B)**.

**Figure 4:**
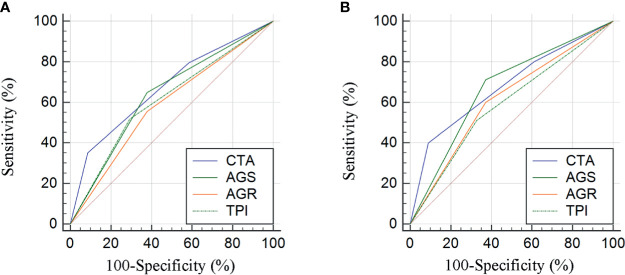
Comparison of area under curves (AUCs) based on the results from receiver operator characteristic (ROC) analyses for AGR, AGS, TPI, and CTA grade in OS and DFS prediction **(A, B)**.

Stratified by CTA grades, 35 (31.2%) patients were classified as grade 1, while grades 2 and 3 contained 53 (47.3%) and 24 (21.4%) patients, respectively (**Table 3**). Patients of CTA grade 1 were considered to have longer OS than grade 2/3 (*p* = 0.047). Moreover, the patients in grade 1 were associated with significantly increased hemoglobin and ALB, but decreased GLB (*p* = 0.002; 0.022; <0.001). According to the Kaplan–Meier survival curves, the patients of grade 1 possessed the highest OS and DFS compared to grade 2/3 (both *p* < 0.001, **Figures 3A, B**). Subsequently, the ROC analyses were applied to evaluate the OS and DFS predictive performance of CTA, AGS, AGR, and TPI (**Figures 4A, B**). The AUC of CTA grade was highest in the above predictive factors (0.674 for OS and 0.681 for DFS; **Table 4**).

**Table 3:** Baseline characteristics according to the subgroups classified by CTA.

Characteristic	CTA			p value
	Grade 1	Grade 2	Grade 3	
	No. (%)	No. (%)	No. (%)	
Total patients	35 (31.2)	53 (47.3)	24 (21.4)	
Age, year, mean (SD)	62.03 (11.67)	67.38 (8.59)	67.04 (12.33)	0.053
Age categorized				0.361
≤65	21 (60.0)	24 (45.3)	11 (45.8)	
>65	14 (40.0)	29 (54.7)	13 (54.2)	
Gender				0.002
Male	35 (100.0)	45 (84.9)	17 (70.8)	
Female	0 (0.0)	8 (15.1)	7 (29.2)	
BMI [kg/m^2^, mean (SD)]	24.72 (2.69)	23.64 (3.08)	21.46 (2.57)	<0.001
BMI categorized, kg/m^2^				0.026
<18.5	1 (2.9)	1 (1.9)	3 (12.5)	
18.5~23.9	13 (37.1)	31 (58.5)	16 (66.7)	
24.0~26.9	15 (42.9)	17 (32.1)	5 (20.8)	
≥27.0	6 (17.1)	4 (7.5)	0 (0.0)	
CCI				0.815
≤2	20 (57.1)	29 (54.7)	15 (62.5)	
>2	15 (42.9)	53 (47.3)	9 (37.5)	
Adjuvant chemotherapy				0.572
No	29 (82.9)	41 (77.4)	21 (87.5)	
Yes	6 (17.1)	12 (22.6)	3 (12.5)	
T-stage				0.488
T1	16 (45.7)	22 (41.5)	8 (33.3)	
T2	7 (20.0)	11 (20.8)	3 (12.5)	
T3	7 (20.0)	8 (15.1)	9 (37.5)	
T4	5 (14.3)	12 (22.6)	4 (16.7)	
N-stage				0.316
N0	32 (91.4)	42 (79.2)	21 (87.5)	
N1	3 (8.6)	11 (20.8)	3 (12.5)	
M-stage				0.814
M0	33 (94.3)	51 (96.2)	24 (100.0)	
M1	2 (5.7)	2 (3.8)	0 (0.0)	
Grade				0.565
Low grade	2 (5.7)	3 (5.7)	3 (12.5)	
High grade	33 (94.3)	50 (94.3)	21 (87.5)	
Hemoglobin [g/L, mean (SD)]	130.29 (17.90)	119.81 (18.63)	111.88 (24.47)	0.002
ALB [g/L, mean (SD)]	40.91 (3.78)	38.19 (5.87)	36.25 (9.88)	0.022
GLB [U/L, mean (SD)]	22.94 (2.07)	26.04 (4.11)	27.42 (5.20)	<0.001
AGR [mean, (SD)]	1.80 (0.24)	1.51 (0.34)	1.33 (0.31)	<0.001
Hospital time (days)	33.58 (9.63)	31.91 (8.88)	37.33 (8.16)	0.053
Survival time (months)	45.80 (25.07)	43.98 (27.49)	29.50 (26.29)	0.047

BMI, body mass index; CCI, Charlson Comorbidity Index; SD, standard deviation; AJCC, American Joint Committee on Cancer; ALB, albumin; GLB, globulin; AGR, albumin to globulin ratio; TPI, total psoas index; AGS, albumin–globulin score; TNM, tumor-node metastasis; CTA, the combination of total psoas index and albumin–globulin score.

**Table 4:** The AUCs of TPI, AGR, AGS, and CTA for OS and DFS prediction based on the ROC results.

Characteristics	OS			DFS		
	AUC	95% CI	p value	AUC	95% CI	p value
TPI	0.613	0.508–0.718	0.035	0.591	0.483–0.700	0.098
AGR	0.588	0.482–0.694	0.103	0.613	0.507–0.720	0.037
AGS	0.634	0.531–0.738	0.011	0.669	0.567–0.771	0.001
CTA	0.674	0.574–0.774	0.001	0.681	0.577–0.784	0.001

AGR, albumin to globulin ratio; TPI, total psoas index; AGS, albumin–globulin score; CTA, the combination of total psoas index and albumin–globulin score; AUC, area under curve; ROC, receiver operator characteristic; OS, overall survival; DFS, disease-free survival; CI, confidence interval.

### Construction and Verification of Nomogram

Univariate and multivariate regression analyses were conducted to determine the independent predictors for OS and DFS of BCa patients after RC. As shown in **Table 5**, the clinical variables including age, T stage, N stage, AGR, AGS, and CTA grade were prognostic factors for OS from the results of univariate regression analysis. However, only age (*p* = 0.013), T stage (*p* < 0.001), and CTA grade (*p* = 0.010) were considered as the predictors for OS based on the multivariate analysis. For DFS, the results from univariate regression analysis indicated that age, TNM stage, AGR, AGS, and CTA grade were related with the OS of BCa patients (**Table 6**). Moreover, the results from multivariate analysis excluded age, N stage, AGR, and AGS grade from independent predictors, which only included T stage (*p* < 0.001), M stage (*p* = 0.022), and CTA grade (*p* = 0.001) (**Table 6**).

**Table 5:** Univariate and multivariate analyses of factors associated with overall survival (OS).

Characteristics	Univariate analysis		Multivariate analysis	
	HR (95% CI)	*p* value	HR (95% CI)	*p* value
Age categorized, years				
≤65	Reference		Reference	
>65	2.073 (1.184–3.629)	0.011	2.075 (1.170–3.679)	0.013
Gender				
Male	Reference		Reference	
Female	0.593 (0.236–1.192)	0.267	–	0.154
CCI				
≤2	Reference		Reference	
>2	1.052 (0.614–1.802)	0.853	–	0.587
Adjuvant chemotherapy				
No	Reference		Reference	
Yes	1.518 (0.810–2.843)	0.189	–	0.145
T-stage		<0.001		<0.001
T1	Reference		Reference	
T2	2.698 (1.160–6.271)	0.021	3.284 (1.404–7.682)	0.006
T3	4.197 (1.933–9.112)	<0.001	3.095 (1.390–6.890)	0.006
T4	5.634 (2.565–12.376)	<0.001	6.778 (3.029–15.166)	<0.001
N-stage				
N0	Reference		Reference	
N1	3.208 (1.716–5.995)	<0.001	–	0.202
M-stage				
M0	Reference		Reference	
M1	2.663 (0.826–8.585)	0.101	–	0. 081
TPI				
Low (0)	Reference		Reference	
High (1)	0.583 (0.340–1.000)	0.050	–	0.951
AGR				
≤1.52	Reference		Reference	
>1.52	0.566 (0.329–0.975)	0.040	–	0. 963
AGS				
Low (0)	Reference		Reference	
High (1/2)	2.304 (1.303–4.075)	0.004	–	0.916
CTA		0.001		0.003
Grade 1	Reference		Reference	
Grade 2	1.511 (0.739–3.091)	0.258	1.491 (0.723–3.076)	0.279
Grade 3	3.617 (1.711–7.644)	0.001	3.697 (1.683–8.125)	0.001

CCI, Charlson Comorbidity Index; AGR, albumin to globulin ratio; TPI, total psoas index; AGS, albumin–globulin score; TNM, tumor-node-metastasis; CTA, the combination of total psoas index and albumin–globulin score; HR, hazard ratio; CI, confidence interval.

**Table 6:** Univariate and multivariate analyses of factors associated with disease-free survival (DFS).

Characteristics	Univariate analysis		Multivariate analysis	
	HR (95% CI)	*p* value	HR (95% CI)	*p* value
Age categorized, years				
≤65	Reference		Reference	
>65	1.883 (1.029–3.445)	0.040	–	0.125
Gender				
Male	Reference		Reference	
Female	0.552 (0.197–1.545)	0.258	–	0.179
CCI				
≤2	Reference		Reference	
>2	0.887 (0.488–1.612)	0.694	–	0.996
Adjuvant chemotherapy				
No	Reference		Reference	
Yes	1.710 (0.882–3.319)	0.108	–	0.116
T-stage		<0.001		<0.001
T1	Reference		Reference	
T2	4.706 (1.704–12.996)	0.003	5.663 (2.034–15.768)	0.001
T3	7.072 (2.731–18.314)	<0.001	4.871 (1.842–12.880)	0.001
T4	9.050 (3.422–23.938)	<0.001	11.286 (4.181–30.465)	<0.001
N-stage				
N0	Reference		Reference	
N1	3.737 (1.927–7.246)	<0.001	–	0.159
M-stage				
M0	Reference		Reference	
M1	3.326 (1.024–10.801)	0.046	4.792 (1.259–18.235)	0.022
TPI				
Low (0)	Reference		Reference	
High (1)	0.596 (0.330-1.076)	0.086	–	0.948
AGR				
≤1.52	Reference		Reference	
>1.52	0.492 (0.271–0.893)	0.020	–	0.947
AGS				
Low (0)	Reference		Reference	
High (1/2)	2.937 (1.539–5.605)	0.001	–	0.797
CTA		<0.001		0.001
Grade 1	Reference		Reference	
Grade 2	1.373 (0.616–3.060)	0.439	1.353 (0.603–3.033)	0.464
Grade 3	4.056 (1.818–9.049)	0.001	4.313 (1.849–10.063)	0.001

CCI, Charlson Comorbidity Index; AGR, albumin to globulin ratio; TPI, total psoas index; AGS, albumin–globulin score; TNM, tumor-node metastasis; CTA, the combination of total psoas index and albumin–globulin score; HR, hazard ratio; CI, confidence interval.

**Figure 5:**
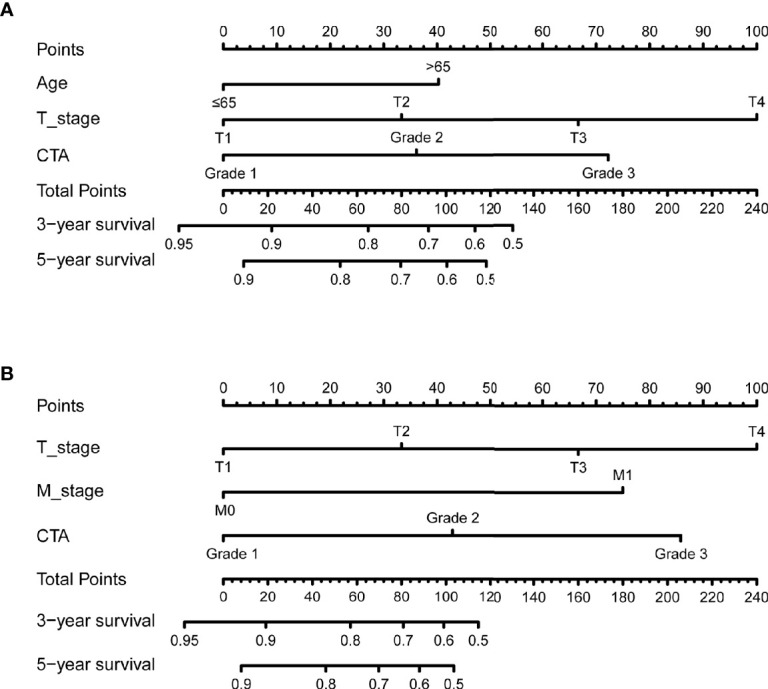
The 3- and 5-year nomogram model for OS and DFS of bladder cancer (BCa) patients after radical cystectomy (RC) based on CTA grade **(A, B)**.

**Figure 6:**
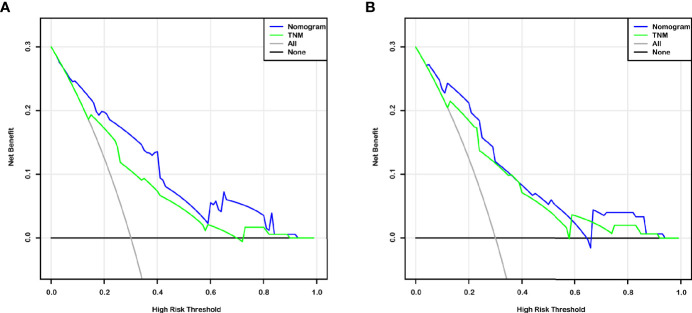
Decision curve analysis (DCA) based on nomogram and TNM-stage for OS and DFS prediction of BCa patients after RC **(A, B)**.

The independent predictors from multivariate analysis results were applied in the establishment of nomograms, which included age, T stage, and CTA grade for the OS nomogram and T stage, M stage, and CTA grade for the DFS nomogram. The 3- and 5-year OS and DFS nomograms are demonstrated in **Figures 5A, B**, and the “total points” calculated by every corresponding factor point could be used to assess the probability. Simultaneously, the calibration curve which could be developed to evaluate the predictive accuracy of nomogram was measured in this study. As shown in **Supplementary Figure S1**, the results indicated that the nomogram possessed a good agreement with the actual observation. The evaluations were conducted through the bootstrap with 1,000 resamples. ROC analyses based on nomograms also proved the effective prediction of the 3-year (**Supplementary Figure S2A**) and 5-year OS and DFS (**Supplementary Figure S2B**). The AUC of nomograms was higher than that in the TNM stage from ROC analyses, indicating the better prediction for OS (0.830 vs. 0.744) and DFS (0.821 vs. 0.770) (**Supplementary Figures S3A, B**). In addition, the DCA curve was further used to compare the predictive performance of the nomogram with TNM stage, which proves that the nomogram contained the better predictive function of OS and DFS (**Figures 6A, B**).

## Discussion

This work introduced a novel predictive index by the combination of ALB, GLB, and the situation of skeletal muscle. The grade of CTA was proved to be an effective indicator for the comprehensive evaluation of the systematic nutritional and inflammatory status in BCa patients. Meanwhile, the impact of CTA grade on long-term outcomes of patients after RC was investigated in detail and exhibited the accurate predictive performance for OS and DFS as the independent risk factor. The nomogram based on CTA grade provided a visualized predictive model for clinicians to optimize individualized therapy.

The AGS that originated in ALB and GLB was regarded as the indicator reflecting not only nutritional status but also the systematic inflammatory responses. Serum ALB, the protein generated in liver, is the indicator measuring the protein synthetic ability *in vivo*. In addition, it remains an independent risk factor of various cancers (18, 19). It was reported that the capability of regulating DNA replication and alleviating oxidative damage contributes to the anticancer performance of ALB (13). For serum GLB, its ability to reflect systemic inflammatory and immune status promotes its application in medicine. It contains kinds of immunoglobulins, such as C-reactive protein, immunoglobulin G (IgG), and immunoglobulin A (IgA) (20, 21). Moreover, it functioned well in prognosis prediction of pancreatic cancer and liver cancer (10, 22). AGS grade, as the prognostic predictor, has been estimated in the survival of patients with lung and liver cancer (10, 13). Li et al. (13) found that AGS grade was related with TNM stage and accurately predicted OS and DFS in 365 operable patients. The same results were obtained in another study of 613 liver cancer patients, and it was found that AGS was associated with liver function, TNM stage, postoperative survival, etc. (10). In this work, we firstly investigated the relationship between AGS grade with postoperative survival of BCa patients. It was evidenced that AGS grade could reflect the OS and DFS of BCa patents after RC and the predictive performance was better than AGR.

The effect of TPI, reflecting the sarcopenia, on postoperative survival was estimated in our work. In the present study, we found that TPI was a risk factor for OS and DFS of BCa patients after RC. The results were consistent with previous studies (15, 23). As the marker of skeletal muscle, TPI was related with mortality and survival time (15). Skeletal muscle was related with immune senescence, and the weak status was considered as the predictor of progression of liver cancer (10). Unexpectedly, the multivariable Cox results were not consistent with our previous report that TPI was the independent survival risk factor (15), which would attribute to the multicollinearity of risk factors included in this study. For a comprehensive evaluation of the systematic nutritional and inflammatory status of BCa patients, a novel index (CTA grade) was introduced by combination of TPI and AGS. The CTA grade would take the systemic inflammation and nutritional status into consideration for prognosis prediction. The results from ROC analyses proved that the CTA grade contained the highest AUC compared with AGS, TPI, and AGR (0.674 for OS and 0.681 for DFS). Li et al. (10) reported that the combination of ALB, GLB, and skeletal muscle status functioned well in predicting OS and RFS in postoperative liver cancer patients, which was consistent with our finding. Recently, the nomogram has been used as the predictive model for various cancers for its features of sample and visualization (24). In addition, the nomogram based on CTA grade was established for 3- and 5-year OS and DFS prediction. This study also verified the predictive performance of the nomogram through DCA and ROC analyses, which indicates the better function of survival prediction than the TNM stage. Our work provided clinicians with a directive and effective tool to optimize therapy for BCa.

To our knowledge, this work was the first attempt to combine ALB, GLB, and skeletal muscle status for long-term outcome prediction of BCa patients after RC. Different from the single independent factor, CTA grade took nutritional status and inflammatory response into consideration at once. In practical, clinical variables were interrelated with each other and an effective tool was desired to integrate risk factors. We expended the predicting values of ALB and GLB by integrating with TPI and finally provided clinicians with CTA grade for patients’ survival estimation. The novel index accurately and effectively predicted the OS and DFS of postoperative BCa patients based on the pro-operative evaluation of systematic nutritional status and inflammatory responses. At the same time, we simplified the predictive process by the conduction of the nomogram without additional workload for clinicians and medical cost for patients.

This study firstly revealed the relationship between CTA grade with long-term outcomes of BCa patients after RC. However, several limitations still needed to be properly addressed in the next stage. Firstly, this work was retrospectively designed, which should be verified by the prospective studies. Secondly, the data were retrieved from one medical center which could not represent other regions. Third, the number of samples should be expended to increase the credibility of results. Therefore, the prospective international multicenter studies were necessary to conduct for the assessment of our results.

## Conclusion

This work proved the novel index which integrated skeletal muscle mass and AGS, which was significantly associated with the OS and DFS of BCa patients after RC. The evaluation of CTA grade effectively predicted the long-term outcomes of patients, which was further visualized by the nomogram. Our results assist clinicians to optimize individualized therapy. Appropriate preoperative physical exercise and nutritional support would contribute to improving survival of BCa patients after RC.

## Data Availability Statement

The datasets used and/or analyzed during the current study are available from the corresponding author on reasonable request.

## Ethics Statement

The studies involving human participants were reviewed and approved by the Medical Ethics Committee of Shanghai Tenth People’s Hospital. The patients/participants provided their written informed consent to participate in this study.

## Author Contributions

(I) Conception and design: KW, WM, JG and BP. (II)Administrative support: TX and BP. (III) Provision of study materials or patients: JG and JX. (IV) Collection and assembly of data: YG, JN, and JX. (V) Data analysis and interpretation: KW and HZ. All authors contributed to the article and approved the submitted version.

## Funding

This work was supported by the National Natural Science Foundation of China (Grant No. 81870517, 32070646); Shanghai Association for Science and Technology Commission (Grant No. 19140905700); Climbing Talent Projects of Shanghai Tenth People’s Hospital (No. 2018SYPDRC046); and the Fundamental Research Funds for the Central Universities (No. 22120180586).

## Conflict of Interest

The authors declare that the research was conducted in the absence of any commercial or financial relationships that could be construed as a potential conflict of interest.

## Publisher’s Note

All claims expressed in this article are solely those of the authors and do not necessarily represent those of their affiliated organizations, or those of the publisher, the editors and the reviewers. Any product that may be evaluated in this article, or claim that may be made by its manufacturer, is not guaranteed or endorsed by the publisher.

## Supplementary Material

Supplementary Figure 1 Calibration plot of the 3- and 5-year OS **(A, C)** and DFS **(B, D)** nomogram.

Supplementary Figure 2 ROC curves based on the nanogram for the 3- and 5-year OS and DFS prediction **(A, B)**.

Supplementary Figure 3 ROC curves based on the nanogram and TNM-stage for the OS and DFS prediction **(A, B)**.

## References

[1] Dobruch J Daneshmand S Fisch M Lotan Y Noon AP Resnick MJ. Gender and Bladder Cancer: A Collaborative Review of Etiology, Biology, and Outcomes. Eur Urol (2016) 69(2):300–10. doi: 10.1016/j.eururo.2015.08.037 26346676

[2] Grayson M. Bladder Cancer. Nature (2017) 551(7679):S33–S. doi: 10.1038/551S33a 29117156

[3] Siegel RL Miller KD Jemal A. Cancer Statistics, 2020. CA Cancer J Clin (2020) 70(1):7–30. doi: 10.3322/caac.21590 31912902

[4] Parekh DJ Reis IM Castle EP Gonzalgo ML Woods ME Svatek RS. Robot-Assisted Radical Cystectomy Versus Open Radical Cystectomy in Patients With Bladder Cancer (RAZOR): An Open-Label, Randomised, Phase 3, Non-Inferiority Trial. Lancet (Lond Engl) (2018) 391(10139):2525–36. doi: 10.1016/S0140-6736(18)30996-6 29976469

[5] Wang K Mao W Shi H Wang G Yin L Xie J. Marital Status Impacts Survival in Patients With Upper Tract Urothelial Carcinoma: A Population-Based, Propensity-Matched Study. Trans Androl Urol (2020) 9(4):1611–29. doi: 10.21037/tau-20-605 PMC7475671 32944523

[6] Mao W Xie J Wu Y Wu Z Wang K Shi H. Cost-Effectiveness Analysis of Two Kinds of Bladder Cancer Urinary Diversion: Studer Versus Bricker. Trans Androl Urol (2020) 9(3):1113–9. doi: 10.21037/tau.2020.03.46 PMC7354334 32676395

[7] Wang K Shi H Mao W Yin L Wang G Fan D. Role of Lymph Node Dissection in Radical Cystectomy. Oncol Lett (2020) 20(1):409–19. doi: 10.3892/ol.2020.11563 PMC7285989 32565966

[8] Mandaliya H Jones M Oldmeadow C Nordman II. Prognostic Biomarkers in Stage IV non-Small Cell Lung Cancer (NSCLC): Neutrophil to Lymphocyte Ratio (NLR), Lymphocyte to Monocyte Ratio (LMR), Platelet to Lymphocyte Ratio (PLR) and Advanced Lung Cancer Inflammation Index (ALI). Trans Lung Cancer Res (2019) 8(6):886–94. doi: 10.21037/tlcr.2019.11.16 PMC6976360 32010567

[9] Dupré A Malik HZ. Inflammation and Cancer: What a Surgical Oncologist Should Know. Eur J Surg Oncol J Eur Soc Surg Oncol Br Assoc Surg Oncol (2018) 44(5):566–70. doi: 10.1016/j.ejso.2018.02.209 29530345

[10] Li H Dai J Lan T Liu H Wang J Cai B. Combination of Albumin-Globulin Score and Skeletal Muscle Index Predicts Long-Term Outcomes of Intrahepatic Cholangiocarcinoma Patients After Curative Resection. Clin Nutr (2021) 40(6):3891–900. doi: 10.1016/j.clnu.2021.04.038 34134006

[11] Liu C Wang W Meng X Sun B Cong Y Liu J. Albumin/globulin Ratio is Negatively Correlated With PD-1 and CD25 mRNA Levels in Breast Cancer Patients. OncoTargets Ther (2018) 11:2131–9. doi: 10.2147/OTT.S159481 PMC5905531 29899663

[12] Li Q Meng X Liang L Xu Y Cai G Cai S. High Preoperative Serum Globulin in Rectal Cancer Treated With Neoadjunctive Chemoradiation Therapy is a Risk Factor for Poor Outcome. Am J Cancer Res (2015) 5(9):2856–64. PMC4633912 26609491

[13] Li X Qin S Sun X Liu D Zhang B Xiao G. Prognostic Significance of Albumin-Globulin Score in Patients With Operable Non-Small-Cell Lung Cancer. Ann Surg Oncol (2018) 25(12):3647–59. doi: 10.1245/s10434-018-6715-z 30229416

[14] Cruz-Jentoft AJ Bahat G Bauer J Boirie Y Bruyère O Cederholm T. Sarcopenia: Revised European Consensus on Definition and Diagnosis. Age Ageing (2019) 48(1):16–31. doi: 10.1093/ageing/afy169 30312372 PMC6322506

[15] Mao W Ma B Wang K Wu J Xu B Geng J. Sarcopenia Predicts Prognosis of Bladder Cancer Patients After Radical Cystectomy: A Study Based on the Chinese Population. Clin Trans Med (2020) 10(2):e105–e. doi: 10.1002/ctm2.105 PMC7403655 32535994

[16] Cruz-Jentoft AJ Sayer AA. Sarcopenia. Lancet (Lond Engl) (2019) 393(10191):2636–46. doi: 10.1016/S0140-6736(19)31138-9 31171417

[17] Kasahara R Kawahara T Ohtake S Saitoh Y Tsutsumi S Teranishi J-i. A Low Psoas Muscle Index Before Treatment Can Predict a Poorer Prognosis in Advanced Bladder Cancer Patients Who Receive Gemcitabine and Nedaplatin Therapy. BioMed Res Int (2017) 2017:7981549. doi: 10.1155/2017/7981549 28497065 PMC5406717

[18] Liu Z Jin K Guo M Long J Liu L Liu C. Prognostic Value of the CRP/Alb Ratio, A Novel Inflammation-Based Score in Pancreatic Cancer. Ann Surg Oncol (2017) 24(2):561–8. doi: 10.1245/s10434-016-5579-3 27650825

[19] Itami Y Miyake M Tatsumi Y Gotoh D Hori S Morizawa Y. Preoperative Predictive Factors Focused on Inflammation-, Nutrition-, and Muscle-Status in Patients With Upper Urinary Tract Urothelial Carcinoma Undergoing Nephroureterectomy. Int J Clin Oncol (2019) 24(5):533–45. doi: 10.1007/s10147-018-01381-y 30604161

[20] Castro-Dopico T Clatworthy MR. IgG and Fcγ Receptors in Intestinal Immunity and Inflammation. Front Immunol (2019) 10:805. doi: 10.3389/fimmu.2019.00805 31031776 PMC6473071

[21] Lamb YN Syed YY Dhillon S. Immune Globulin Subcutaneous (Human) 20% (Hizentra(®)): A Review in Chronic Inflammatory Demyelinating Polyneuropathy. CNS Drugs (2019) 33(8):831–8. doi: 10.1007/s40263-019-00655-x 31347096

[22] Feng L Gu S Wang P Chen H Chen Z Meng Z. Pretreatment Values of Bilirubin and Albumin are Not Prognostic Predictors in Patients With Advanced Pancreatic Cancer. Cancer Med (2018) 7(12):5943–51. doi: 10.1002/cam4.1848 PMC6308037 30474926

[23] Mayr R Gierth M Zeman F Reiffen M Seeger P Wezel F. Sarcopenia as a Comorbidity-Independent Predictor of Survival Following Radical Cystectomy for Bladder Cancer. J Cachexia Sarcopenia Muscle (2018) 9(3):505–13. doi: 10.1002/jcsm.12279 PMC5989852 29479839

[24] Wang K Wu Z Wang G Shi H Xie J Yin L. Survival Nomogram for Patients With Bone Metastatic Renal Cell Carcinoma: A Population-Based Study. Int Braz J Urol (2021) 47(2):333–49. doi: 10.1590/S1677-5538.IBJU.2020.0195 PMC7857761 33284535

