# Supplementary figures and images for: Combination of Total Psoas Index and Albumin–Globulin Score for the Prognosis Prediction of Bladder Cancer Patients After Radical Cystectomy: A Population-Based Study
Source: Front Oncol. 2021 Sep 20;11:724536. doi: 10.3389/fonc.2021.724536 (PMC8488353; doi:10.3389/fonc.2021.724536)

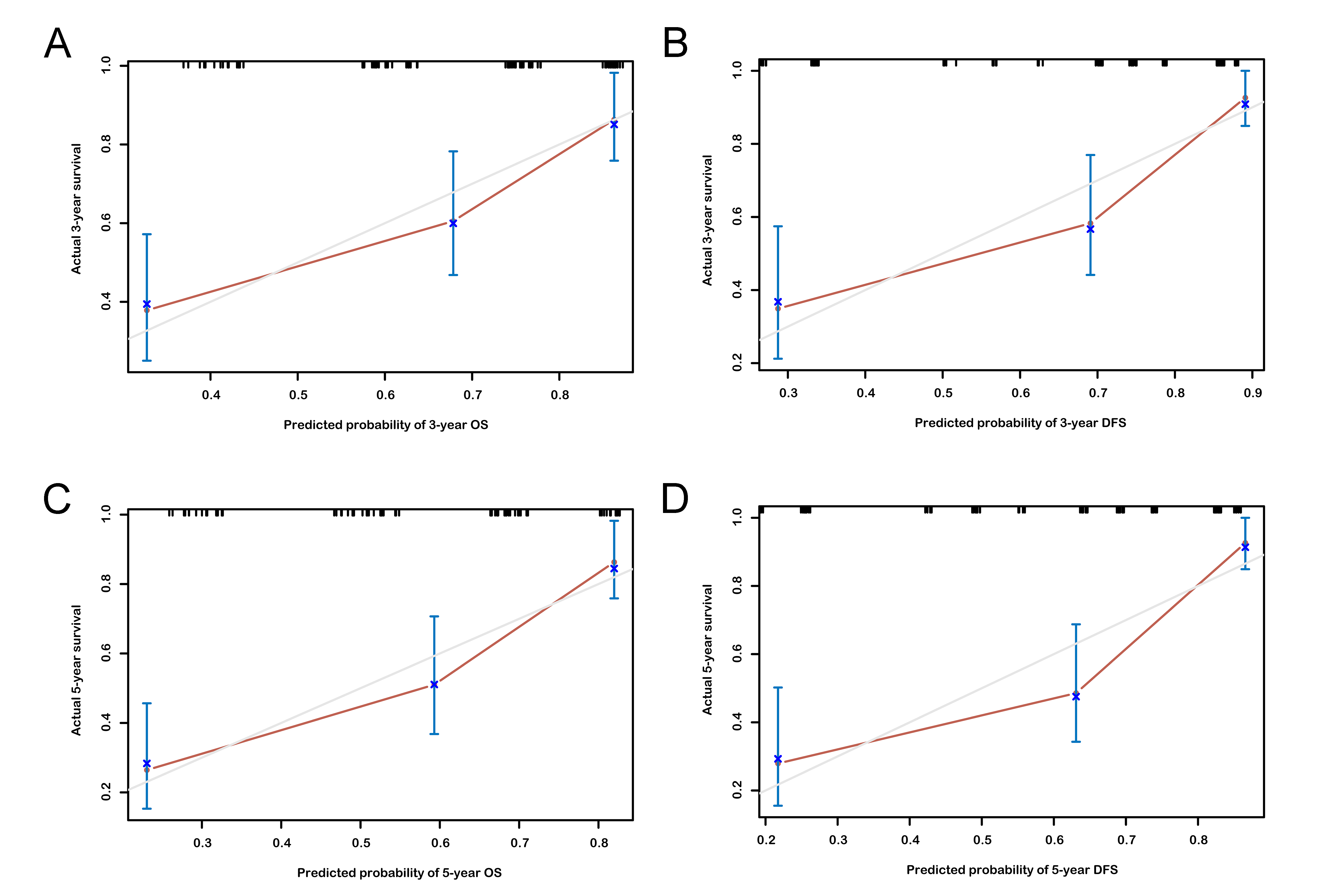

Supplement: Supplementary Figure 1 — Calibration plot of the 3- and 5-year OS (A, C) and DFS (B, D) nomogram. [file Image_1.tif]

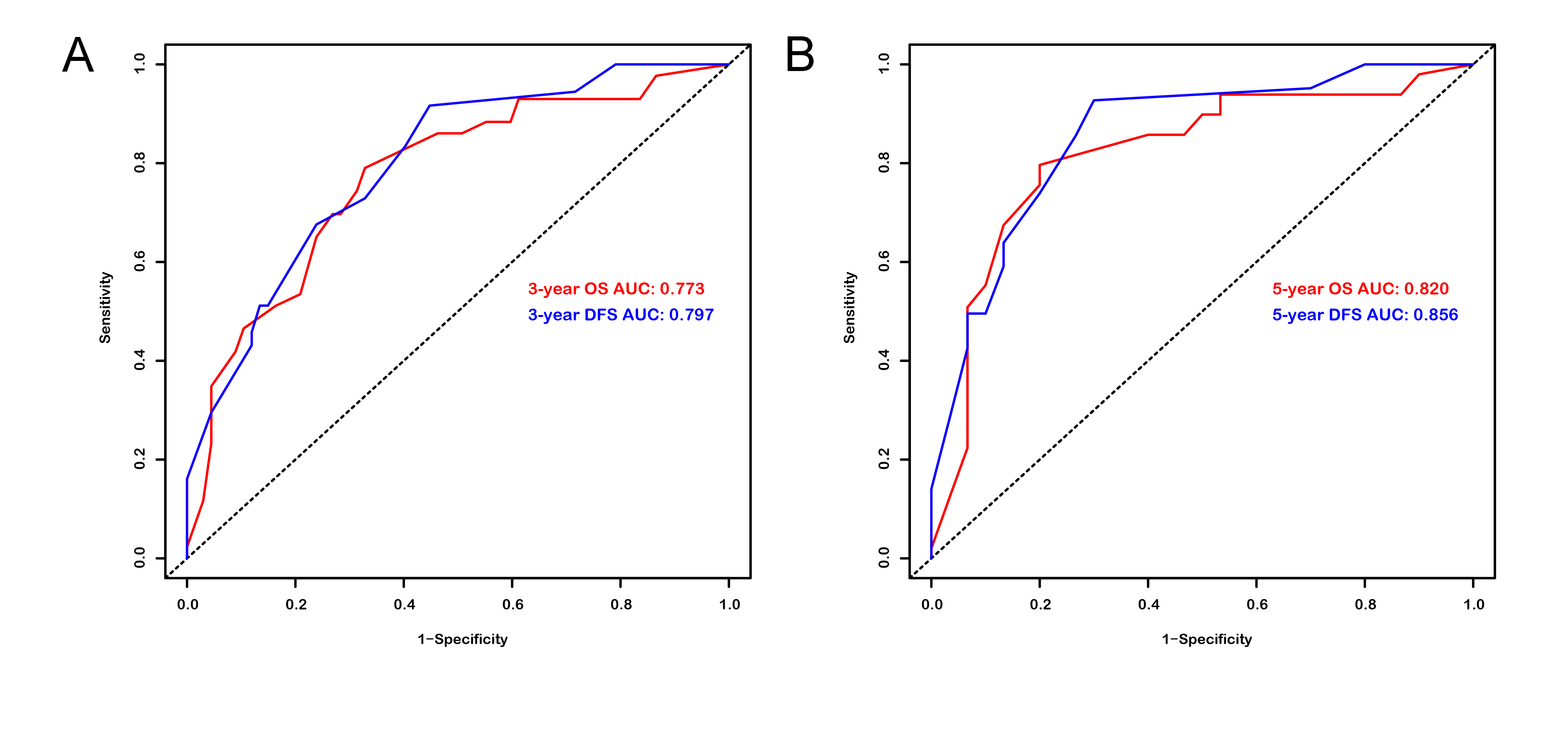

Supplement: Supplementary Figure 2 — ROC curves based on the nanogram for the 3- and 5-year OS and DFS prediction (A, B). [file Image_2.tif]

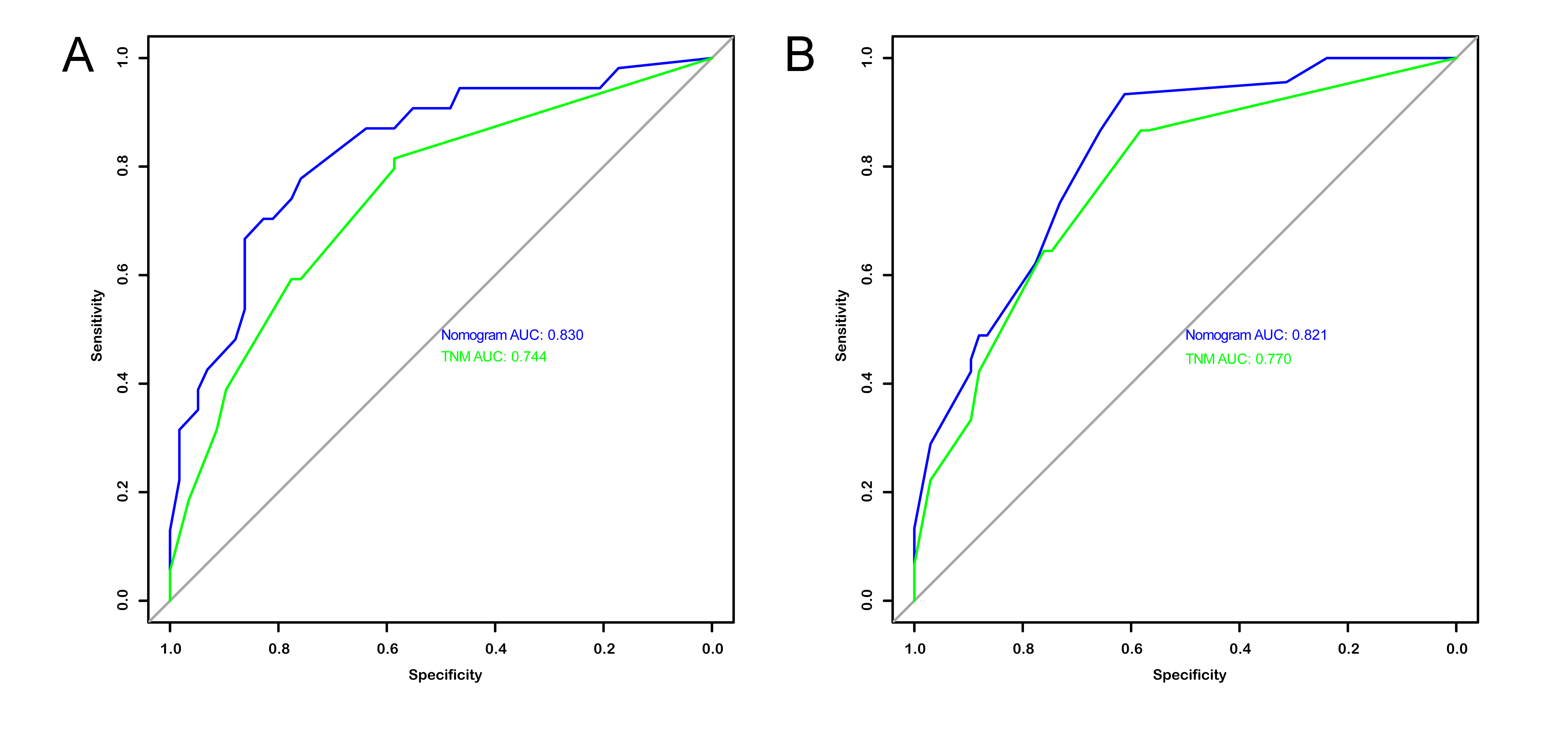

Supplement: Supplementary Figure 3 — ROC curves based on the nanogram and TNM-stage for the OS and DFS prediction (A, B). [file Image_3.tif]
